# Chemical composition, cholinesterase, and α-glucosidase inhibitory activity of the essential oils of some Iranian native *Salvia* species

**DOI:** 10.1186/s12906-023-04004-w

**Published:** 2023-06-03

**Authors:** Houra Jazayeri Gharehbagh, Masoud Ebrahimi, Farid Dabaghian, Somayeh Mojtabavi, Roshanak Hariri, Mina Saeedi, Mohammad Ali Faramarzi, Mahnaz Khanavi

**Affiliations:** 1 Department of Pharmacognosy, Faculty of Pharmacy, Tehran University of Medical Sciences, Tehran, Iran; 2 Department of Pharmaceutical Biotechnology, Faculty of Pharmacy, Tehran University of Medical Sciences, Tehran, Iran; 3 Department of Medicinal Chemistry, Faculty of Pharmacy, Tehran University of Medical Sciences, Tehran, Iran; 4 Medicinal Plants Research Center, Faculty of Pharmacy, Tehran University of Medical Sciences, Tehran, Iran; 5 Persian Medicine and Pharmacy Research Center, Tehran University of Medical Sciences, Tehran, Iran; 6 Faculty of Land and Food Systems, University of British Columbia, Vancouver, BC Canada

**Keywords:** Alzheimer's disease, Cholinesterase, Diabetes mellitus, Essential oil, α-Glucosidase, *Salvia*

## Abstract

**Background:**

The plants from *Salvia* genus contain widely distributed species which have been used in folk medicine as well as pharmaceutical and food industries.

**Methods:**

The chemical composition of 12 native Iranian *Salvia* species (14 plants) was identified using gas chromatography-mass spectrometry (GC–MS). Also, the inhibitory activity of all essential oils (EOs) was evaluated toward α-glucosidase and two types of cholinesterase (ChE) using spectrophotometric methods. The in vitro α-glucosidase inhibition assay was performed by the determination of* p*-nitrophenol (*p*NP) obtained from the enzymatic dissociation of* p*-nitrophenol-α-D-glucopyranoside (*p*NPG) as the substrate. In vitro ChE inhibitory assay was conducted based on the modified Ellman’s procedure using the measurement of 5-thio-2-nitrobenzoic acid produced from the hydrolysis of thiocholine derivatives as the substrate, in the presence of acetylcholinesterase (AChE) and butyrylcholinesterase (BChE).

**Results:**

Totally, 139 compounds were detected and caryophyllene oxide and *trans*-β-caryophyllene were the most abundant compounds in all EOs. The yield of EOs extracted from the plants were also calculated in the range of 0.06 to 0.96% w/w. Herein, α-glucosidase inhibitory activity of 8 EOs was reported for the first time and among all, *S. spinosa* L. was found to be the most potent inhibitor (90.5 inhibition at 500 μg/mL). Also, the ChE inhibitory activity of 8 species was reported for the first time and our results showed that the BChE inhibitory effect of all EOs was more potent than that of AChE. The ChE inhibition assay indicated that *S. mirzayanii* Rech.f. & Esfand. collected from Shiraz was the most potent inhibitor (72.68% and 40.6% at the concentration of 500 μg/mL, toward AChE and BChE, respectively).

**Conclusions:**

It seems that native *Salvia* species of Iran could be considered in the development of anti-diabetic and anti-Alzheimer's disease supplements.

## Background

Plants and their extracts, essential oils (EOs), and secondary metabolites have been widely used in the pharmaceutical, food, cosmetic, and perfume industries due to valuable medicinal, flavoring, and preserving properties. Among them, *Salvia* species have attracted a lot of attention [1]; for example, *Salvia officinalis* L. (sage) is used for food preservation, particularly meat and cheese [2]. In this respect, *Salvia sclarea* L., *Salvia hispanica* L., and *Salvia divinorum* Epling & Játiva are cultivated in many parts of the world due to their commercial interests [3, 4].

*Salvia* is one of the most important and largest genera of the Lamiaceae family containing approximately 900 distinct species worldwide (tropical, temperate, and arctic regions) [5] and widely distributed in Iran. Out of 58 different species existing in Iran, 17 are endemic [6]. The extracts and EOs of *Salvia* have depicted a wide range of biological activities including antibacterial, carminative, diuretic, spasmolytic, anti-inflammatory, antioxidant, anti-cancer, anti-diabetic, anxiolytic, and sedative properties. Also, the genus has long been considered in folk medicine to deal with different ailments such as epilepsy, cancer, malaria, bronchitis, tuberculosis, hepatitis, anti-diabetic, dementia, and dysmenorrhea [6-10]. Furthermore, *Salvia* plants have been extensively used for different therapeutic purposes in Persian medicine. For example, *S. officinalis* has been used as a diuretic, carminative, wound healing, and asthma-treating agent [11-13]. Today, the sale and production of sage have resulted in significant income benefits for several Asian nations due to its several applications in aromatherapy and promoting general health as well as food industry [14].

Diabetes Mellitus (DM) is an endocrine disease which can impair carbohydrate metabolism due to insulin deficiency or insulin resistance [15]. The global diabetes prevalence is worrying, and it is estimated that over 700 million people will suffer from the disease, by the year 2045 [16]. Sulfonylureas, biguanides, and other drugs possessing various mechanisms of action are used to treat type 2 diabetes mellitus (T2DM), which is the most common type of DM. Delaying the uptake of glucose by inhibiting α-glucosidase, has been an efficient therapeutic tool for the treatment of T2DM [17]. α-Glucosidase is the key enzyme which is located in the brush border of the intestine, catalyses dietary carbohydrates to glucose monomers and prepares them for absorption. Thus, post-prandial blood glucose can be controlled by inhibiting the enzyme [3]. However, continuous use of approved drugs causes adverse effects such as hypoglycemia, nausea, and dizziness [18, 19]. Recently, many studies have focused on plant-derived natural products which are safer and more affordable than the common medications. Also, various plants have been used as anti-diabetic agents in folk medicine, their EOs and extracts can be regarded as extremely valuable natural resources for the treatment of DM [20, 21]. In an in vivo study, *S. officinalis* EO exhibited anti-diabetic properties by reducing blood glucose up to 60% and elevating stored glycogen in the liver up to 43.7% [22]. Assaggaf et al. evaluated the chemical composition and α-glucosidase inhibitory activity of *S. officinalis* EO in the full flowering stage which inhibited the enzyme with an IC_50_ = 22.24 μg/mL, compared to acarbose as a standard (IC_50_ = 12.31 μg/mL) [23].

*Salvia* species have also been linked to neuroprotective characteristics. Generally, the small size and lipophilicity of EOs constituents allow them to easily pass the blood–brain barrier and therefore could be suggested as a potential strategy for the treatment of neurodegenerative disease [24]. Nowadays, approximately 50 million people suffer from Alzheimer’s disease (AD) worldwide and a three-fold increase in the incidence of the disease is estimated by 2050 [25]. The cholinesterases (ChEs) including AChE and BChE are responsible for the hydrolysis of acetylcholine (ACh) in the brain. Thus, reduction of the level of ACh can be terminated by the inhibition of ChEs. Currently, FDA-approved ChE inhibitors such as donepezil, rivastigmine, and galantamine are important medicines for controlling the symptoms of AD. The EOs of medicinal plants are rich sources of valuable phytochemicals which have been widely considered for the treatment of various neurodegenerative diseases such as AD [26]. In this regard, based on a clinical study, the EOs of *Salvia lavandulifolia* Vahl. and *S. officinalis* significantly improved memory performance and thereby, could be considered in aromatherapy [27]. Furthermore, *S. lavandulifolia* EO was found to be a selective AChE inhibitor with an IC_50_ value of 3 µg/mL while inhibiting BChE by 22% at 0.5 mg/mL [28]. Also, *S. lavandulifolia* EO reduced the activity of AChE in the striatum of rats, however, not in their hippocampus or cortex [29]. Moreover, in vitro evaluation of *Salvia potentillifolia* Boiss. & Heldr. ex Benth. EO indicated inhibitory activity against the BChE by 65.7% inhibition at the concentration of 200 µg/mL and the corresponding activity on AChE was obtained as 21.9% inhibition at the same concentration, compared with galantamine as a reference (75.5% and 81.4% inhibition at 200 μM, respectively) [30].

Unlike synthetic substances, which are often based on a single active component, EOs include a variety of compounds that interact synergistically or additively with each other to either reduce the risk of drug resistance or boost the effectiveness of the treatment [31]. Several studies have reported the chemical composition of different species of *Salvia* genus EOs and α-pinene, β-pinene, germacrene D, spathulenol, bicyclogermacrene, 1,8-cineole, camphor, borneol, α-thujone and β-thujone, thymol, caryophyllene, and caryophyllene oxide have been commonly determined as the most prominent components [6, 32].

The scientific research of numerous plant species to find new natural bioactive agents is a time-consuming and resource-intensive procedure. As a result, researchers are now receiving assistance in their quest to identify active pharmaceutical ingredients in medicinal plants, which were previously used to treat illnesses in a simple and cost-effective manner. In this study, constituents of EOs of 14 plants from 12 Iranian native *Salvia* species were investigated, and they were evaluated for their α-glucosidase and ChE inhibitory activity to develop dual natural anti-diabetic and anti-AD agents as AD has been considered as type 3 diabetes and the role of insulin resistance in inducing impaired brain glucose metabolism, neurodegeneration, and cognitive impairment has been comprehensively discussed in the literature [33].

## Methods

### Chemicals

α-Glucosidase (from *Saccharomyces cerevisiae*; EC3.2.1.20, 20 U/mg), acetylcholinesterase (AChE, E.C. 3.1.1.7, Type V-S, lyophilized powder, from electric eel, 1000 unit), butyrylcholinesterase (BChE, E.C. 3.1.1.8, from equine serum), *p*-nitrophenyl α-D-glucopyranoside (*p*-NPG), 5,5′-dithio-bis-(2-nitrobenzoic acid) (DTNB), acetylthiocholine iodide (ATCI), and butyrylthiocholine iodide (BTCI) were provided from Sigma-Aldrich.

### Plant material

**Fig. 1:**
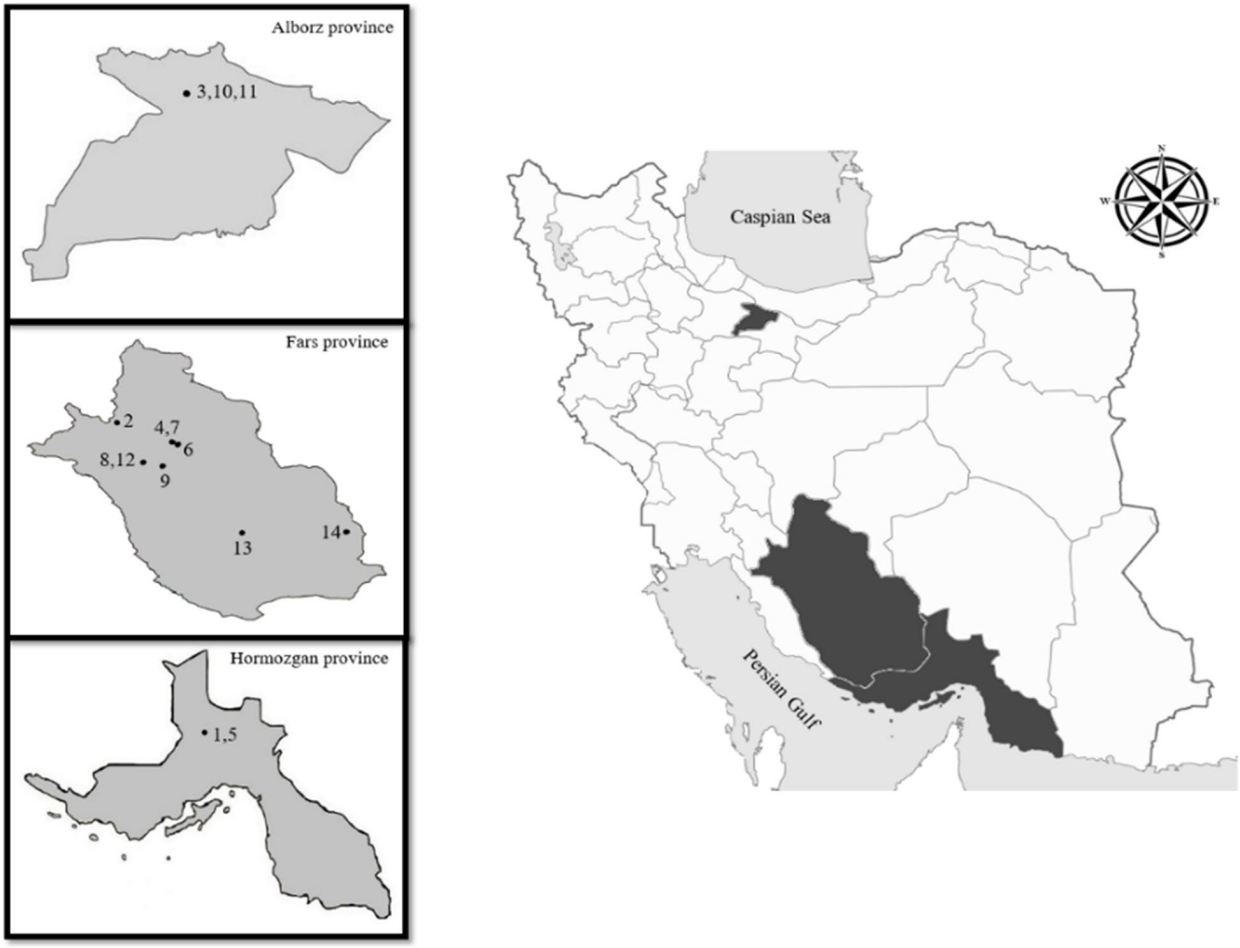
Geographical distribution of  14 studied plants from *Salvia* genus

Aerial parts of 14 native Iranian medicinal plants from 12 distinct species of the *Salvia* genus were collected from different parts of Iran during the flowering stage. After verification by the botanist Sedighe Khademian at Shiraz University of Medical Sciences, voucher specimens were deposited in the herbarium of the Faculty of Pharmacy, Tehran University of Medical Sciences, Tehran, Iran (Table 1, Fig. 1). Comparing the identified herbarium and flora specimens with the identified plant species enabled for reliable plant identification. Experimental research and field studies on plants (either cultivated or wild), including the collection of plant material, complies with relevant institutional, national, and international guidelines and legislation.

**Table 1:** EOs obtained from aerial parts of some *Salvia* spp., collected from different parts of Iran

Code	Species	Collecting site	Wild/ Cultivated	Latitude	Longitude	Altitude (m)	Voucher No	EO yield (%) w/w
1	*Salvia sharifii* Rech.f. & Esfand	Sarchahan village, Hormozgan province	Wild	28.05° N	55.87° E	850	7102-TEH	0.07
2	*Salvia sclarea* L	Kahkaraan village, Sepidan, Fars province	Wild	30.36° N	52.07° E	2400	7106-TEH	0.12
3	*Salvia verticillata* L	Khodkavand village, Taleghan, Alborz province	Wild	36.14° N	50.83° E	2150	7074-TEH	0.09
4	*Salvia syriaca* L	Pir-sabz-ali village, Kamfiruz, Marvdasht Fars province	Wild	30.50° N	52.09° E	1900	7104-TEH	0.11
5	*Salvia santolinifolia* Boiss	Sarchahan village, Hormozgan province	Wild	28.05° N	55.87° E	800	7091-TEH	0.13
6	*Salvia reuterana* Boiss	Kamfiruz, Marvdasht, Fars province	Wild	30.31° N	52.19° E	1800	7086-TEH	0.06
7	*Salvia multicaulis* vahl	Pir-sabz-ali village, Kamfiruz, Marvdasht Fars province	Wild	30.50° N	52.09° E	1900	7110-TEH	0.26
8	*Salvia spinosa* L	Shiraz, Fars province	Cultivated	29.59° N	52.58° E	1500	7108-TEH	0.11
9	*Salvia palaestina* Benth	Kaftarak village, Fars province	Wild	29.57° N	52.69° E	1500	7103-TEH	0.20
10	*Salvia virgata* Jacq	Gelyard village, Taleghan, Alborz province	Wild	36.15° N	50.84° E	2150	7073-TEH	0.08
11	*Salvia hypoleuca* Benth	Gelyard village, Taleghan, Alborz province	Wild	36.15° N	50.84° E	2150	7075-TEH	0.36
12	*Salvia mirzayanii* Rech.f. & Esfand	Shiraz, Fars province	Cultivated	29.59° N	52.58° E	1500	7114-TEH	0.47
13	*Salvia mirzayanii* Rech.f. & Esfand	Goldamcheh village, Jahrom, Fars province	Wild	28.64° N	53.51° E	1100	7113-TEH	0.92
14	*Salvia mirzayanii* Rech.f. & Esfand	Mazayjan village, Darab, Fars province	Wild	30.29° N	53.80° E	850	7112-TEH	0.65

### Extraction of the essential oils

The aerial parts of plants were collected and dried at room temperature away from direct sunlight. To isolate the corresponding EO, each milled dried plant (100 g) was hydro-distilled for 4 h using a Clevenger-type apparatus according to European Pharmacopoeia (2020). Anhydrous sodium sulfate was added to the isolated EO to remove the water. All EOs were stored in a dark sealed vial at 4 °C for further experiments. The yield of extraction was reported as w/w%.

### GC–MS analysis

GC–MS analysis was performed on a 7890B Agilent gas chromatograph including a DB-5 column (60 cm, 0.25 µ) and a 5977A Agilent mass spectrometer. 1 μL of diluted samples (with ethyl acetate) was injected into the injection site. The temperature program was scheduled as follows: the initial temperature of the oven was 40˚C (held for 7 min) and programmed to reach 140˚C with a rate of 10 ˚C /min, eventually reached 250˚C with a rate of 3˚C /min and held for 7 min at this temperature. Helium with 99.99% purity was utilized as a carrier gas (flow rate: 1 mL/min). Also, the ionization voltage of the detector was set at 70 eV. To determine the components, normal alkanes (C_7_–C_21_) were injected in the same manner to compare calculated retention indices with those in authentic references. For more accurate identification, the mass spectra of each compound were reconciled with the NIST database [34] and Adams's book [35].

### α-Glucosidase inhibition assay

The α-glucosidase inhibitory activity of EOs was evaluated based on the previously described method [20] using α-glucosidase (from *Saccharomyces cerevisiae*).

### Cholinesterase inhibition assay

Inhibitory activity against AChE (E.C. 3.1.1.7, Type V-S, lyophilized powder, from electric eel, 1000 unit) and BChE (E.C. 3.1.1.8, from equine serum) was performed using modified Ellman's method [36].

### Statistical analysis

The GraphPad Prism software was used to carry out statistical analysis. Data comparisons were performed by one-way analysis of variance (ANOVA) with Tukey’s multiple comparisons as the post-hoc test. *P* values < 0.05 were considered statistically significant.

## Results

### Yield of isolation of essential oils

The yield of isolation of EOs from each species was reported in Table 1. They were obtained in the range of 0.06–0.92 w/w%. The lowest and highest values were related to *S. reuterana* and *S. mirzayanii* (Jahrom region), respectively.

### Chemical composition of essential oils

According to the GC–MS analysis, 139 components were identified in the isolated EOs from 14 plants as recorded in Table 2. They were categorized into monoterpenes, sesquiterpenes, oxygenated diterpenes, carbonyl compounds, alcohols, acids, esters, alkanes, and phenolic compounds. As reported in Table 2, sesquiterpenes were the major compounds in the series of *Salvia* spp. EOs. However, in the case of *S. multicaulis*, monoterpenes were the most abundant components.

**Table 2:** Chemical composition of EOs (%) of some native Iranian *Salvia* spp.^a^

Entry	Compounds	RI^b^	RI^c^	1	2	3	4	5	6	7	8	9	10	11	12	13	14	Class
1	α-Tricyclene	917	917	-	-	-	-	-	-	0.39	-	-	-	-	-	-	-	MH
2	α-Thujene	923	923	0.37	-	-	-	0.05	-	-	-	0.11	-	-	-	-	-	MH
3	α-Pinene	934	934	2.57	0.09	0.21	0.31	**4.56**	-	**9.43**	1.11	0.07	-	4.76	-	0.05	0.1	MH
4	Camphene	952	952	0.81	-	-	0.58	1.00	-	5.23	-	-	-	0.63	-	-	-	MH
5	Sabinene	973	973	1.29	-	0.5	-	-	-	-	-	-	-	1.06	-	0.08	0.08	MH
6	1-Octen-3-ol	974	974	-	0.34	-	-	0.59	0.25	0.34	0.59	0.44	1.64	-	-	-	-	Alcohol
7	β-Pinene	981	981	2.57	0.1	0.86	-	0.95	-	1.61	1.3	0.05	1.18	4.46	-	0.14	0.25	MH
8	β-Myrcene	986	986	0.22	0.8	0.5	-	0.13	-	0.49	-	-	0.31	-	0.8	0.3	0.81	MH
9	dehydro-1,8-Cineole	993	991	-	-	-	-	-	-	-	-	-	-	-	-	0.88	0.26	OM
10	Mesitylene	994	992	-	0.5	0.35	**9.13**	0.2	0.38	-	1.22	0.47	1.14	-	-	-	-	Others
11	2-δ-Carene	999	994	-	-	0.17	-	-	-	-	-	-	-	-	-	-	-	MH
12	α-Phellandrene	1006	1003	-	-	0.02	-	0.15	-	-	-	-	-	-	-	-	-	MH
13	α-Terpinene	1018	1015	0.48	-	0.09	-	0.29	-	0.22	-	0.08	-	0.18	-	0.11	0.11	MH
14	*p*-Cymene	1024	1022	**3.71**	-	0.28	-	2.07	1.07	2.83	-	1.18	2.60	-	-	0.13	-	MH
15	Limonene	1032	1029	2.68	-	1.34	-	2.23	-	4.91	1.08	-	2.32	0.42	1.32	1.07	1.53	MH
16	Sylvestrene	1033	1031	-	0.65	-	-	-	-	-	-	-	-	-	-	-	-	MH
17	1,8-Cineole	1037	1034	0.83	-	0.72	1.85	-	-	1.1	0.41	0.01	0.97	0.34	1.97	1.78	2.6	OM
18	*cis*-β-Ocimene	1043	1040	-	0.79	0.03	-	0.12	0.23	-	-	0.05	0.22	-	0.67	0.19	0.62	MH
19	β-Phellandrene	1049	1046	-	-	0.83	-	0.29	-	-	-	-	-	-	-	-	-	MH
20	γ-Terpinene	1060	1057	**3.71**	-	0.15	-	0.36	-	1.36	-	0.35	1.4	0.31	-	0.15	0.15	MH
21	Terpinolene	1090	1088	0.69	0.33	0.07	-	0.25	0.09	0.15	0.62	-	0.25	-	0.45	0.38	0.55	MH
22	*p*-Cymenene	1092	1089	-	-	-	-	0.32	-	0.05	-	-	-	-	-	-	-	MH
23	Linalool	1099	1097	2.67	**8.41**	0.3	2.38	0.52	**5.20**	0.16	**20.94**	2.18	**7.67**	0.64	5.14	2.16	4.75	OM
24	Butanoic acid, 2-methyl, 3-methylbutyl ester	1101	1100	0.81	-	-	-	-	1.22	-	-	0.16	-	-	-	-	-	Ester
25	Butanoic acid, 2-methyl, 2-methylbutyl ester	1103	1100	0.72	-	-	-	-	0.7	-	0.92	-	-	-	-	-	-	Ester
26	Nonanal	1110	1107	-	-	0.09	-	-	-	0.05	-	-	0.28	-	-	-	-	CC
27	Fenchol	1127	1122	-	-	-	-	1.31	-	0.09	-	-	-	-	-	-	-	OM
28	α-Campholenal	1132	1126	0.48	-	-	-	1.48	-	0.13	-	-	-	-	-	-	-	OM
29	*trans*-Pinocarveol	1151	1146	1.2	-	0.1	-	3.32	-	1.42	1.12	-	2.26	0.29	-	-	-	OM
30	Camphor	1160	1156	-	-	-	-	0.63	-	4.28	-	-	-	-	-	-	-	OM
31	Octanoic acid	1165	1161	-	-	-	-	-	-	-	0.86	0.03	-	-	-	-	-	Acid
32	Pinocarvone	1171	1165	0.84	-	0.03	-	1.39	-	0.22	-	-	-	-	-	-	-	OM
33	δ-Terpineol	1179	1170	-	-	0.09	-	-	-	-	1.42	-	0.35	-	0.9	1.04	0.59	OM
34	Borneol	1185	1176	0.28	-	0.29	-	**7.68**	0.07	**12.52**	-	0.03	-	1.13	-	-	-	OM
35	Terpinen-4-ol	1191	1184	2.32	0.04	0.25	-	2.69	-	3.02	0.78	0.04	0.56	0.42	0.21	0.45	0.35	OM
36	α-Terpineol	1205	1198	0.61	3.53	0.21	0.39	3.64	0.72	0.18	1.97	0.09	3.56	0.23	3.53	3.41	3.97	OM
37	Myrtenol	1209	1202	0.64	-	-	-	1.77	-	0.94	-	-	-	-	-	-	0.14	OM
38	Myrtenal	1210	1204	1.04	-	-	-	-	-	-	0.58	-	-	-	-	-	-	OM
39	*trans*-Carveol	1227	1218	-	-	-	-	0.92	-	-	-	-	-	-	-	0.66	-	OM
40	Nerol	1228	1221	-	0.91	-	-	0.31	0.29	0.17	0.81	0.05	0.6	-	0.74	-	0.89	OM
41	Butanoic acid, 2-methyl, hexyl ester	1234	1234	-	-	-	-	-	0.27	-	1.29	0.03	-	-	-	-	-	Ester
42	Butanoic acid, 3-methyl, hexyl ester	1242	1242	0.31	-	0.02	-	-	0.29	0.08	1.54	0.03	-	-	-	-	-	Ester
43	Geraniol	1249	1246	0.17	2.27	0.07	3.1	-	0.68	0.31	1.1	0.09	-	-	2.37	1.47	2.68	OM
44	Linalyl acetate	1254	1249	-	3.41	-	-	-	-	-	-	-	**6.68**	-	1.56	0.54	1.43	OM
45	Decanol	1270	1269	-	-	-	-	0.48	-	-	-	-	0.26	-	0.23	0.61	0.32	Alcohol
46	Bornyl acetate	1288	1279	-	-	0.15	0.93	1.32	-	0.11	-	-	-	-	-	-	-	OM
47	Thymol	1290	1286	3.00	0.71	0.06	2.44	2.28	1.52	0.14	0.09	0.19	0.14	-	1.98	1.22	2.36	PC
48	Carvacrol	1296	1287	2.84	0.77	0.07	3.80	0.69	1.20	**5.66**	0.2	0.07	-	-	1.73	2.28	1.89	PC
49	δ-Elemene	1346	1338	-	-	0.06	1.24	-	0.52	-	1.26	0.31	-	3.2	0.63	0.5	0.53	SH
50	α-Terpinyl acetate	1353	1344	-	-	-	**7.72**	0.12	-	-	0.52	0.03	4.35	0.29	**7.53**	**10.56**	**9.45**	OM
51	Decanoic acid	1354	1349	0.58	-	0.05	-	-	0.36	-	1.36	-	-	-	-	-	-	Acid
52	α-Cubebene	1358	1350	0.12	0.76	0.4	0.52	-	0.18	-	-	0.78	-	-	-	-	-	SH
53	Eugenol	1359	1351	-	-	-	0.9	1.56	-	0.09	-	-	-	-	2.25	1.16	0.6	PC
54	Neryl acetate	1371	1362	-	1.42	-	-	-	0.11	1.13	0.58	-	1.19	-	1.17	0.71	1.91	OM
55	Hexanoic acid, hexyl ester	1382	1370	0.09	-	-	-	-	-	-	2.12	-	-	-	-	-	-	Ester
56	α-Ylangene	1386	1375	-	-	-	-	0.65	-	-	-	0.08	-	-	-	-	-	SH
57	α-Copaene	1389	1377	1.01	**5.42**	1.06	2.58	2.37	-	3.38	-	5.22	-	0.77	-	0.64	0.22	SH
58	Geranyl acetate	1391	1381	-	2.91	-	-	-	-	0.22	-	-	-	-	-	-	-	OM
59	β-Maaliene	1392	1382	-	-	-	-	-	-	0.06	-	-	-	-	-	-	-	SH
60	*trans*-β-Damascenone	1393	1382	0.65	0.36	-	0.52	-	0.43	-	0.9	0.16	0.18	-	-	-	-	OM
61	β-Cubebene	1394	1383	-	-	-	-	-	0.83	-	0.58	-	0.26	-	-	-	-	SH
62	β-Bourbonene	1403	1388	0.89	0.62	6.05	-	-	-	-	-	-	-	1.3	-	-	-	SH
63	β-Elemene	1404	1391	-	-	-	1.18	-	2.28	-	3.09	-	1.84	-	1.83	1.75	1.67	SH
64	*cis*-β-Caryophyllene	1422	1409	-	-	-	-	-	0.23	0.37	0.67	-	-	-	-	-	-	SH
65	α-Gurjunene	1428	1413	-	-	**9.27**	-	0.15	1.89	1.58	**4.00**	-	0.52	-	1.66	1.85	1.33	SH
66	*trans*-β-Caryophyllene	1441	1430	1.18	3.00	**17.14**	1.64	0.97	2.84	**19.02**	3.33	**13.37**	1.56	**24.12**	0.73	0.98	0.72	SH
67	β-Copaene	1448	1435	-	-	1.56	-	-	-	-	-	0.42	-	-	-	-	-	SH
68	β-Gurjunene	1451	1436	-	-	-	-	0.07	-	1	0.65	-	-	0.39	-	-	-	SH
69	Neryl acetone	1457	1436	0.44	0.31	-	0.39	1.48	0.29	-	-	-	0.7	-	-	-	-	CC
70	α-Guaiene	1459	1441	-	-	0.44	-	-	-	-	-	0.34	0.94	-	-	2.75	1.15	SH
71	Aromandendrene	1460	1445	0.11	-	0.71	-	0.61	-	1.14	0.96	0.46	0.49	0.39	1.17	0.83	1.22	SH
72	*cis*-Muurola-3,5-diene	1467	1450	-	0.07	-	-	-	-	-	-	0.15	-	-	-	0.51	0.37	SH
73	α-Humulene	1476	1462	0.12	0.18	**8.28**	-	0.25	0.59	1.76	1.35	1.62	0.24	1.91	-	0.86	0.39	SH
74	Alloaromadendrene	1480	1465	-	-	2.33	-	0.47	0.14	0.09	-	0.39	-	-	-	-	-	SH
75	9-*epi*-Caryophyllene	1482	1466	-	-	-	-	0.47	-	-	-	-	-	3.73	-	-	-	SH
76	*cis*-Muurola-4(15),5-diene	1483	1467	-	-	-	-	-	-	-	-	-	-	-	0.3	0.68	0.47	SH
77	Cadina-1(6),4-diene	1488	1477	-	-	-	-	-	-	-	0.4	-	-	-	0.35	-	0.47	SH
78	γ-Gurjunene	1492	1477	-	-	2.43	-	-	1.49	0.25	-	-	-	-	-	-	-	SH
79	γ-Muurolene	1493	1479	1.07	0.22	0.27	-	3.33	-	-	1.25	0.7	0.19	0.62	1.23	1.14	1.05	SH
80	α-Elemene	1495	1479	-	-	-	-	-	-	-	-	-	-	-	0.58	-	0.43	SH
81	Germacrene D	1501	1485	0.73	1.91	**13.78**	2.58	0.29	0.81	-	**5.77**	**16.33**	0.24	**9.66**	0.74	0.48	0.47	SH
82	β-Selinene	1503	1490	0.5	0.25	-	-	-	1.64	-	1.19	0.75	0.16	0.68	0.97	0.97	0.93	SH
83	δ-Selinene	1509	1493	-	0.22	0.86	-	1.77	0.25	0.12	0.67	-	0.43	-	1.24	1.33	1.11	SH
84	γ-Amorphene	1510	1496	-	-	0.43	0.37	0.12	0.18	0.08	-	-	1.08	-	-	-	-	SH
85	2-Tridecanone	1511	1496	0.5	-	-	-	-	-	-	-	-	-	-	-	-	-	CC
86	Viridiflorene	1512	1497	-	-	-	-	-	-	0.52	-	-	-	-	-	-	-	SH
87	α-Selinene	1513	1498	-	-	-	-	0.83	-	0.08	0.76	-	-	-	3.55	0.63	-	SH
88	α-Muurolene	1517	1500	-	-	-	-	0.95	-	0.14	-	-	-	-	-	4.75	3.85	SH
89	Bicyclogermacrene	1521	1500	0.12	-	2.34	1.88	-	0.61	-	3.78	**8.67**	3.55	**15.49**	3.13	2.02	2.19	SH
90	γ-Cadinene	1528	1514	0.25	1.59	0.41	-	1.79	0.38	0.22	1.11	0.09	-	0.92	1.69	-	2.29	SH
91	δ-Cadinene	1532	1523	0.47	-	1.35	5.63	**3.67**	0.27	0.64	0.43	3.11	2.98	-	**7.74**	**11.72**	**8.57**	SH
92	*trans*-Calamenene	1535	1526	0.26	0.64	0.81	0.99	1.67	-	0.1	0.65	0.34	-	-	-	-	-	SH
93	*trans*-γ-Bisabolene	1545	1531	-	-	0.31	-	-	-	-	-	-	-	-	-	-	-	SH
94	*trans*-Cadina-1(2),4-diene	1548	1535	-	0.33	-	-	0.41	-	0.03	-	0.2	-	-	0.23	0.47	0.34	SH
95	Liguloxide	1549	1536	-	-	-	-	-	-	-	-	-	0.23	-	0.39	-	0.33	OS
96	α-Cadinene	1551	1539	-	-	0.08	-	0.29	-	-	-	0.23	0.18	-	0.4	1.25	0.64	SH
97	α-Calacorene	1556	1542	-	1.07	0.14	0.68	1.25	-	-	-	0.4	-	-	-	-	-	SH
98	Elemicin	1559	1550	0.28	-	-	-	0.07	0.23	-	-	0.05	-	-	-	-	-	Others
99	β-Calacorene	1565	1559	-	-	0.16	-	0.12	-	-	-	-	-	-	-	-	-	SH
100	Dodecanoic acid	1572	1567	1.51	-	-	-	-	0.41	-	0.11	0.42	-	-	-	-	-	Acid
101	1,5-Epoxysalvial-4(14)-ene	1588	1569	1.14	2.88	-	-	-	-	-	-	0.67	-	-	-	-	-	OS
102	Ledol	1590	1574	-	-	0.13	-	0.13	-	0.1	-	-	-	-	0.45	-	0.35	OS
103	Germacrene D-4-ol	1596	1576	-	-	-	-	-	-	-	-	-	-	-	3.83	-	3.19	OS
104	Spathulenol	1597	1578	**9.47**	4.32	5.12	**17.62**	1.31	3.78	0.94	3.03	7.48	**9.55**	**8.51**	4.50	**6.53**	4.27	OS
105	Caryophyllene oxide	1605	1584	**21.11**	**22.26**	5.90	**6.37**	0.91	**8.99**	3.82	**3.97**	**7.84**	**6.62**	6.41	0.38	0.53	0.4	OS
106	Globulol	1607	1585	-	-	-	-	0.43	-	-	-	-	-	-	-	0.28	0.25	OS
107	β-Copaen-4-α-ol	1608	1591	-	-	-	1.49	-	-	-	-	-	-	-	-	-	-	OS
108	Viridiflorol	1613	1593	-	-	-	-	-	-	-	-	-	-	0.39	0.63	0.71	0.51	OS
109	Salvial-4(14)-en-1-one	1614	1595	1.82	1.5	0.59	1.49	0.07	1.2	-	0.7	0.59	-	-	-	-	-	OS
110	Widdrol	1622	1599	-	-	0.38	-	-	-	-	-	-	-	-	-	-	-	OS
111	α-*epi*-7-*epi*-5-Eudesmol	1625	1608	-	-	-	-	0.25	-	0.06	-	-	-	-	-	-	0.29	OS
112	Humulene epoxide II	1630	1608	-	-	1.86	-	-	-	0.19	-	0.99	0.54	0.32	-	-	-	OS
113	1,10-di-*epi*-Cubenol	1638	1619	-	-	0.25	-	-	-	-	-	0.7	0.71	-	0.24	-	0.3	OS
114	10-*epi*-γ-Eudesmol	1642	1624	0.58	-	-	-	1.47	-	-	-	-	-	-	-	-	-	OS
115	*epi*-Cubenol	1644	1627	-	0.92	-	-	-	-	-	-	-	-	-	0.85	0.74	0.75	OS
116	Isospathulenol	1645	1629	-	-	0.44	5.09	-	2.48	0.23	0.78	0.56	0.58	1.48	-	-	-	OS
117	Eremoligenol	1650	1631	-	-	-	-	0.61	-	-	-	-	-	0.27	0.45	-	0.39	OS
118	γ-Eudesmol	1654	1632	-	-	-	-	0.75	-	-	0.24	-	-	-	-	-	-	OS
119	*epi*-α-Cadinol	1655	1640	-	-	-	0.64	-	-	-	-	-	1.97	-	1.45	-	-	OS
120	Caryophylla-4(14),8(15)-diene-5-β-ol	1657	1641	1.39	1.28	-	-	-	1.34	1.11	-	1.11	-	-	-	-	-	OS
121	Alloaromadendrene epoxide	1658	1641	-	0.7	-	-	-	-	-	0.44	-	-	-	-	-	-	OS
122	*epi*-α-Muurolol	1660	1642	-	-	0.23	1.68	0.77	-	-	0.21	0.48	2.56	-	4.73	5.36	**5.93**	OS
123	α-Muurolol	1662	1646	-	-	-	-	0.41	-	-	-	-	-	-	-	-	-	OS
124	β-Eudesmol	1674	1651	1.11	1.26	0.21	-	**16.1**	1.04	-	0.35	-	1.61	-	1.64	1.53	1.32	OS
125	α-Cadinol	1676	1654	-	-	1.16	2.75	-	-	-	0.75	1.06	1.36	0.59	**8.17**	**5.76**	**6.37**	OS
126	14-Hydroxy-9-*epi*-(*E*)-Caryophyllene	1682	1664	**4.30**	2.98	0.67	-	-	1.51	0.8	-	1.25	2.16	-	-	-	-	OS
127	Valeranone	1694	1675	-	-	0.25	-	-	-	-	-	-	-	-	-	-	-	OS
128	Cadalene	1699	1677	-	-	-	0.5	0.75	-	-	-	-	-	-	-	-	-	SH
129	Eudesma-4(15),7-dien-1-β-ol	1705	1688	1.9	1.35	1.07	0.73	0.09	-	-	-	-	-	-	-	-	-	OS
130	Shyobunol	1717	1691	-	-	-	-	0.23	-	-	0.35	-	2.62	-	**6.43**	3.39	3.72	OS
131	Heptadecane	1720	1700	0.51	-	-	-	-	0.36	-	0.11	0.52	1.44	-	-	-	-	Alkane
132	Mint sulfide	1761	1741	-	-	0.17	-	0.09	-	-	-	-	-	-	-	-	-	Others
133	Benzyl Benzoate	1777	1760	1.32	0.36	-	3.94	0.12	3.29	0.27	-	0.48	-	-	-	-	-	Ester
134	Octadecane	1800	1800	0.92	0.06	0.08	-	0.09	0.72	0.06	0.06	0.27	1.32	-	-	-	-	Alkane
135	Hexahydrofarnesyl acetone	1845	1835	-	-	-	0.77	-	-	-	0.7	0.67	-	-	-	-	-	CC
136	Nonadecane	1900	1900	0.9	-	0.07	-	0.15	0.61	0.06	-	0.24	0.89	-	-	-	-	Alkane
137	Sclareoloxide	1915	1906	1.32	**11.48**	0.2	-	0.15	**24.56**	-	1.19	1.61	-	1.9	-	-	-	OS
138	Hexadecanoic acid	1962	1951	-	-	0.25	-	1.39	4.75	-	-	3.21	-	-	-	-	-	Acid
139	Phytol	2100	2089	0.98	0.64	0.92	-	0.13	**7.84**	0.64	0.52	2.32	3.13	0.95	-	-	-	OD
	Monoterpenes			30.83	26.02	7.26	17.78	39.87	8.89	52.67	35.24	4.57	36.65	15.16	28.36	26.26	33.22	
	Monoterpene hydrocarbons			19.10	2.76	5.05	0.89	12.77	1.39	26.67	4.11	1.89	8.28	11.82	3.24	2.60	4.20	
	Oxygenated monoterpenes			11.73	23.26	2.21	16.89	27.10	7.50	26.00	31.13	2.68	28.37	3.34	25.12	23.66	29.02	
	Sesquiterpenes			50.97	67.21	89.13	57.65	46.93	60.03	37.83	43.91	78.3	45.17	83.05	62.31	60.94	58.78	
	Sesquiterpene hydrocarbons			6.83	16.28	70.67	19.79	23.25	15.13	30.58	31.90	53.96	14.66	63.18	28.17	36.11	30.41	
	Oxygenated sesquiterpenes			44.14	50.93	18.46	37.86	23.68	44.90	7.25	12.01	24.34	30.51	19.87	34.14	24.83	28.37	
	Oxygenated diterpenes			0.98	0.64	0.92	-	0.13	7.84	0.64	0.52	2.32	3.13	0.95	-	-	-	
	Carbonyl compounds			0.94	0.31	0.09	1.16	1.48	0.29	0.05	0.70	0.67	0.98	-	-	-	-	
	Alcohols			0.00	0.34	-	-	1.07	0.25	0.34	0.59	0.44	1.90	-	0.23	0.61	0.32	
	Acids			2.09	-	0.30	-	1.39	5.52	-	2.33	3.66	-	-	-	-	-	
	Esters			3.25	0.36	0.02	3.94	0.12	5.77	0.35	5.87	0.70	-	-	-	-	-	
	Alkanes			2.33	0.06	0.15	-	0.24	1.69	0.12	0.17	1.03	3.65	-	-	-	-	
	Phenolic compounds			5.84	1.48	0.13	7.14	4.53	2.72	5.89	0.29	0.26	0.14	-	5.96	4.66	4.85	
	Others			0.28	0.50	0.52	9.13	0.36	0.61	-	1.22	0.52	1.14	-	-	-	-	
	Identification			97.51	96.92	98.52	96.80	96.12	93.61	97.89	90.84	92.47	92.76	99.16	96.86	92.47	97.17	

^a^1) *S. sharifii*, 2) *S. sclarea*, 3) *S. verticillata*, 4) *S. syriaca*, 5) *S. santolinifolia*, 6) *S. reuterana*, 7) *S. multicaulis*, 8) *S. spinosa*, 9) *S. palaestina*, 10) *S. virgata*, 11) *S. hypoleuca*, 12) *S. mirzayanii* (Shiraz region) 13) *S. mirzayanii* (Jahrom region) 14) *S. mirzayanii* (Darab region)

^b^Retention indices relative to C_7_- C_21_ n-alkanes on DB-5 column

^c^Retention index from literatures; *MH* Monoterpene Hydrocarbon, *OM* Oxygenated Monoterpene, *CC* Carbonylic Compound, *PC* Phenolic Compounds, *SH* Sesquiterpene Hydrocarbon, *OS* Oxygenated Sesquiterpene, *OD* Oxygenated Diterpene. Bold numbers in the table indicate the predominant compounds of each EO

The GC–MS analysis also revealed that among all EOs components (Table 2), sclareol oxide (24.56%), and *trans*-β-caryophyllene (24.12%) (entries 137 and 66, respectively) were the highest in value. It is worth mentioning that caryophyllene oxide was detected as the main compound in 7 *Salvia* spp. *S. sharifii* and *S. sclarea* EOs contained the corresponding compound as 21.11 and 22.26%, respectively, which were more significant than the others. Moreover, linalool, α-terpineol, *trans*-β-caryophyllene, spathulenol, and caryophyllene oxide were ubiquitous in all isolated EOs.

### Biological activity of essential oils

The percentage inhibition values for α-glucosidase and ChE inhibitory activity of the *Salvia* EOs were reported in Table 3.

**Table 3:** Enzyme inhibitory activity of *Salvia* spp. EOs at the concentration of 500 μg/mL^a^

Plants EO	α-glucosidase (inhibition %)	AChE (inhibition %)	BChE (inhibition %)	SI^c^
*S. sharifii*	86.9 ± 0.1^****^	7.8 ± 0.3^****^	42.0 ± 0.3^****^	5.38
*S. sclarea*	33.4 ± 0.5^****^	1.5 ± 0.1^****^	32.8 ± 0.1^****^	21.86
*S. verticillata*	85.5 ± 0.7^****^	1.1 ± 0.1^****^	25.3 ± 0.6^****^	23
*S. syriaca*	33.7 ± 0.3^****^	15.8 ± 0.5^****^	52.1 ± 0.4^****^	3.29
*S. santolinifolia*	88.4 ± 0.7^*^	2.7 ± 0.2^****^	44.1 ± 0.4^****^	16.33
*S. reuterana*	89.7 ± 0.2^ ns^	1.6 ± 0.1^****^	27.8 ± 0.9^****^	17.37
*S. multicaulis*	85.9 ± 0.8^****^	0.5 ± 0.1^****^	44.1 ± 0.5^****^	88.2
*S. spinosa*	90.5 ± 0.1	1.4 ± 0.2^****^	25.5 ± 0.6^****^	18.21
*S. palaestina*	87.6 ± 1.2^****^	2.1 ± 0.1^****^	7.0 ± 0.1^****^	3.33
*S. virgata*	89.7 ± 0.6^ ns^	1.8 ± 0.1^****^	25.5 ± 0.7^****^	14.16
*S. hypoleuca*	22.7 ± 0.7^****^	2.8 ± 0.2^****^	11.3 ± 0.4^****^	4.03
*S. mirzayanii* (Shiraz)	28.5 ± 1.0^****^	40.6 ± 0.5	72.7 ± 0.1	1.79
*S. mirzayanii* (Jahrom)	NA^b^	30.8 ± 0.6^****^	64.8 ± 0.4^****^	2.10
*S. mirzayanii* (Darab)	25.5 ± 0.4^****^	23.9 ± 0.4^****^	63.0 ± 0.3^****^	2.64
Acarbose	50.2 ± 1.1	-	-	-
Donepezil	-	89.9 ± 0.12	83.0 ± 0.1	-

^a^Reported as (Mean ± SD)

^b^Not Active

^c^SI: Selectivity Index = BChE (% inhibition)/AChE (% inhibition)

Mean comparison of each EO was compared with the value obtained from the most potent EO in each biological activity through one-way ANOVA test followed by Tukey post-hoc multiple comparisons (****p < 0.0001, *p < 0.05, and ns = not significant)

### α-Glucosidase inhibitory activity

It was perceived that 8 EOs expressed a notable inhibitory effect toward α-glucosidase at 500 μg/mL, compared with acarbose. More specifically, *S. spinosa* EO exhibited the strongest activity (90.5% inhibition) and EOs of *S. virgata* and *S. reuterana* were also able to block the enzyme with high percentage inhibition (89.7% inhibition). Additionally, the EOs of *S. mirzayanii* collected from Darab and *S. hypoleuca* showed a weak α-glucosidase inhibitory activity with percentage inhibition values of 25.5%, and 22.7%, respectively, while *S. mirzayanii* from Jahrom displayed no activity.

### Cholinesterase inhibitory activity

In vitro ChE inhibitory assay of the 14 investigated EOs indicated moderate to remarkable activity toward both AChE and BChE at 500 μg/mL, however, they were less active than donepezil (Table 3).

*S. mirzayanii* collected from Shiraz revealed the highest inhibitory effect on AChE and BChE (40.6% and 72.68%, respectively), and *S. mirzayanii* collected from Jahrom (30.81% and 64.76%) and Darab (23.86% and 63.02%), as well as *S. syriaca* (15.8% and 52.1%), showed good activity. *S. verticillata* and *S. multicaulis* had a negligible effect toward AChE (0.5% and 1.1% respectively) and *S. palaestina* and *S. hypoleuca* had no significant inhibitory effect on BChE (7% and 11.3%, respectively). Furthermore, *S. sharifii* (42%), *S. santolinifolia* (44.1%), and *S. multicaulis* (44.1%) were found to be moderate inhibitors of BChE.

Finally, the selectivity index (SI) of the tested EOs in the inhibition of BChE over AChE showed that *S. multicaulis* EO was the most selective BChE inhibitor (SI = 88.2). Meanwhile, the three populations of *S. mirzayanii* EOs exhibited no noticeable selectivity in the inhibition of ChEs.

## Discussion

GC–MS analysis indicated that monoterpenes and sesquiterpenes were the predominant components of EOs. Even though significant components were similar in the isolated EOs, some constituents such as viridiflorene, *trans*-γ-bisabolene, β-copaen-4-α-ol, and valeranone were detected only in the specific species. However, caryophyllene oxide, spathulenol, linalool, α-terpineol, and *trans*-β-caryophyllene were detected in all EOs. Additionally, caryophyllene oxide (0.38–22.26%), *trans*-β-caryophyllene (0.72–24.12%), spathulenol (0.94–17.62%), germacrene D (0–16.33%), and δ-cadinene (0–11.72%) were generally found to be the main components in this series of EOs. Comparing the major components of the studied *Salvia* spp. with those reported in the literature (Table 4), revealed the significant variation in the EOs components of different *Salvia* species. Genetic composition, environmental and climate factors, and developmental stages are important reasons that may contribute to this diversity [4, 37].

**Table 4:** Comparison of major components of studied *Salvia* spp. with those reported in the literature

Entry	Species	Major compounds			Reference
		In this study	Literature	Region	
1	*S. sharifii*	Caryophyllene oxide (21.11%), Spathulenol (9.47%), 14-Hydroxy-9-*epi*-(*E*)-Caryophyllene (4.30%)	Linalool (32.95%), Hexyl isovalerate (15.44%), Hexyl 2‐methyl butanoate (10.99%)	Tunisia	[38]
			Linalool (20.74%), Spathulenol (7.98%), Caryophyllene oxide (4.77%), Isopentyl isovalerate (4.48%)	Hormozgan, Iran	[39]
2	*S. sclarea*	Caryophyllene oxide (22.26%), Sclareoloxide (11.48%), Linalool (8.41%)	Linalool (38.07%), α-Terpineol (13.40%), Geraniol (5.67%)	Lebanon	[40]
			Linalyl acetate (45.51%), Linalool (38.98%), α-Terpineol (5.85%)	Ukraine	[41]
3	*S. verticillata*	*trans*-β-Caryophyllene (17.14%), Germacrene D (13.78%), α-Gurjunene (9.27%), α-Humulene (8.28%)	β-Pinene (30.7%), *p*-Cymene (23.0%), Isopropyl ester of lauric acid (16.8%)	Greece	[42]
			*trans*-β-Caryophyllene (41.0%), α-Humulene (14.0%), Germacrene D (13.0%), Bicyclogermacrene (13.0%)	Ardebil, Iran	[37]
4	*S. syriaca*	Spathulenol (17.62%), Mesitylene (9.13%), α-Terpinyl acetate (7.72%), Caryophyllene oxide (6.37%)	Spathulenol (20.5%), Borneol (17.9%), Bicyclogermacrene (11.1%), Germacrene D (10.7%)	Ardebil, Iran	[43]
			Germacrene-D (21.77%), *trans*-β-ocimene (14.66%), β-Pinene (9.70%)	Turkey	[44]
5	*S. santolinifolia*	β-Eudesmol (16.1%), Borneol (7.68%), α-Pinene (4.56%)	α-Pinene (49.3%), β-Eudesmol (20.0%), Camphene (7.8%), Limonene (7.7%)	Zahedan, Iran	[45]
6	*S. reuterana*	Sclareoloxide (24.56%), Caryophyllene oxide (8.99%), Phytol (7.84%)	Caryophyllene oxide (38.0%), Spathulenol (17.0%), *trans*-β-Caryophyllene (9.67%)	Isfahan, Iran	[37]
			β-Elemene (13.92%), ɑ-Gurjunene (13.7%), Isoaromadendrene epoxide (11.9%)	Shahmirzad, Iran	[46]
7	*S. multicaulis*	*trans*-β-Caryophyllene (19.02%), Borneol (12.52%), α-Pinene (9.43%)	α-Pinene (15.5%), Camphene (10.41%), 1,8-Cineole (13.59%)	Kurdistan, Iran	[47]
			1,8-Cineole (29.35%), α-Pinene (14.49%), Camphor (12.48%)	Tehran, Iran (cultivated)	[48]
8	*S. spinosa*	Linalool (20.94%), Germacrene D (5.77%), α-Gurjunene (4.00%), Caryophyllene oxide (3.97%)	Caryophyllene oxide (63.0%), Spathulenol (23.0%), Linalool (3.9%)	Kerman, Iran	[49]
			Thymol (68.9%), Isopentyl isovalerate (5.3%), Isopentyl 2-methyl (4.1%)	Jordan	[50]
9	*S. palaestina*	Germacrene D (16.33%), *trans*-β-Caryophyllene (13.37%), Bicyclogermacrene (8.67%), Caryophyllene oxide (7.84%)	Germacrene D (26.02%), α-Copaene (18.58%), β-Cubebene (8.75%)	Jordan	[51]
10	*S. virgata*	Spathulenol (9.55%), Linalool (7.67%), Linalyl acetate (6.68%), Caryophyllene oxide (6.62%)	*trans*-β-Caryophyllene (30.0%), δ-Cadinene (16.0%), Caryophyllene oxide (10.0%)	Isfahan, Iran	[37]
			*trans*-β-Caryophyllene (23.1%), Sabinene (18.2%), *cis*-β-Farnesene (12.3%)	Mazandaran, Iran	[52]
11	*S. hypoleuca*	*trans*-β-Caryophyllene (24.12%), Bicyclogermacrene (15.49%), Germacrene D (9.66%), Spathulenol (8.51%)	*trans*-β-Caryophyllene (18.3%), β-Pinene (13.3%), α-Pinene (13.1%)	Tehran, Iran	[53]
			Bicyclogermacrene (15.3%), Viridiflorol (13.3%), Spathulenol (12.5%)	Tehran, Iran	[54]
12	*S. mirzayanii*	α-Cadinol (8.17%), δ-Cadinene (7.74%), α-Terpinyl acetate (7.53%), Shyobunol (6.43%) (Shiraz)	γ-Cadinene (12.5%), Caryophyllene oxide (8.5%), Bicyclogermacrene (7.7%), α-Terpinyl acetate (6.7%)	Lamerd, Iran	[55]
		δ-Cadinene (11.72%), α-Terpinyl acetate (10.56%), Spathulenol (6.53%), α-Cadinol (5.76%) (Jahrom)	1,8-Cineole (41.2%), Linalool acetate (10.7%), α-Terpinyl acetate (5.7%)	Bandar Abbas, Iran	[56]
		α-Terpinyl acetate (9.45%), δ-Cadinene (8.57%), α-Cadinol (6.37%), *epi*-α –Muurolol (5.93%) (Darab)	8-Acetoxy linalool (10.97%), Linalool (9.01%), 1,8-Cineole (8.03%), Linalyl acetate (7.63%)	Hormozgan, Iran	[57]

According to the literature review, α-glucosidase inhibitory activity of *S. syriaca*, *S. spinosa*, *S. virgata*, and *S. sclarea* EOs have been previously recorded. Bahadori et al. reported that the α-glucosidase inhibitory activity of *S. syriaca* EO (IC_50_ = 1.2 mg/mL) was more potent than acarbose (IC_50_ = 9.6 mg/mL) [58] which is in contrary with our findings. In another study, the α-glucosidase inhibitory activity of *S. spinosa* EO was evaluated, which demonstrated moderate inhibitory activity with an IC_50_ value of 43.79 µg/mL, compared with acarbose (IC_50_ = 17.1 µg/mL) [49]. On the contrary, another study indicated that neither *S. virgata* nor *S. sclarea* EOs were active toward α-glucosidase [7].

EOs possessing high amounts of *p*-cymene, borneol, γ-terpinene, and phytol have shown good inhibitory effect on α-glucosidase. For example, *Carum carvi* L. and *Coriandrum sativum* L. EOs containing high amounts of these compounds showed very potent α-glucosidase inhibitory activity, compared with acarbose [59] which is in alignment with our results. Although no study was reported on the inhibitory activity of β-eudesmol, α-humulene, and camphene, which are present in high concentration in the *S. santolinifolia*, *S. verticillata* and *S. multicaulis* EOs, respectively; they may be effective in inducing α-glucosidase inhibitory activity.

AChE accounts for roughly 80% of cholinesterase activity in the normal brain, with BChE making up the remaining 20%. However, in severe AD, AChE activity may decline to 55–67% of baseline levels in particular brain regions, whereas BChE activity rises. Also, BChE may contribute to the accumulation of β-amyloid plaques that occur in the early phases of AD progression [60]. Therefore, nowadays there is a considerable deal of interest in discovering compounds that can precisely inhibit the BChE.

To the best of our knowledge, cholinesterase inhibitory activity of the *S. sharifii*, *S. santolinifolia*, *S. reuterana*, *S. spinosa*, *S. palaestina*, *S. virgata*, *S. hypoleuca*, and *S. mirzayanii* EOs are reported for the first time and those of *S. sclarea*, *S. verticillata*, *S. multicaulis,* and *S. syriaca* EOs were previously investigated [58, 61-63].

Orhan et al. [63] studied the BChE inhibitory activity of two populations of cultivated *S. sclarea* which were exposed to different fertilizers. The EOs of plants treated with organic and chemical fertilizers were able to inhibit the enzyme with the percentage inhibition of 76.0% and 45.1%, respectively at the concentration of 1 mg/mL. However, *S. sclarea* EO exhibited 32.8% inhibition toward BChE at the concentration of 500 μg/mL, in our study. On the other hand, only the plant EO treated with chemical fertilizers were able to inhibit the AChE (11.6%, at 1 mg/mL), while in our study, *S. sclarea* EO inhibited this enzyme less than 10% at 500 μg/mL.

Kunduhoglu et al. reported the cholinesterase inhibitory activity of *S. verticillata* and *S. wiedemannii* EOs. In agreement with our results, *S. verticillata* EO had a weak inhibitory effect on the ChEs (20.4% and 1.8% against AChE and BChE, respectively), however, inconsistent with our results, the EO inhibited the AChE more strongly than the BChE [62].

*S. syriaca* EO demonstrated AChE and BChE inhibitory activity stronger than the reference (galantamine) [58], while compared to other understudied EOs in our survey, *S. syriaca* EO showed significant inhibition on the ChEs, but they couldn't exceed donepezil as the standard.

As reported by Akdeniz et al., *S. multicaulis* EO was able to inhibit AChE and BChE by 25.6% and 71.2%, respectively, at the concentration of 100 μg/mL [61], but the EO inhibited BChE by 44.1% with no inhibitory activity on AChE, in our study (at 500 μg/mL).

According to the study of Loizzo et al. on the EO of *S. leriifolia*, α-pinene and 1,8-cineole showed BChE inhibitory activity with IC_50_ values of 0.87 and 0.93 mM, respectively [64]. Also, good AChE inhibitory activity has been reported for α-pinene, however, 1,8-cineole has not been active [63]. Although these results were in good agreement with ours in some cases, the α-pinene content was not found to be important for inducing desired AChE inhibitory activity in isolated EOs.

According to the Chowdhury and Kumar report, α-terpinyl acetate was introduced as a natural monoterpenoid with potent ChE inhibitory activity (IC_50_ values of 54.7 and 47.5 µM against AChE and BChE, compared with donepezil with IC_50_ values of 0.15 and 5.8 µM, respectively) [65]. These results were also supported by ours indicating that *S. mirzayanii* and *S. syriaca* EOs have α-terpinyl acetate in higher amounts than other EOs, which induced higher ChEs inhibitory properties particularly more potent BChE inhibitory activity.

Orhan et al. showed that geraniol inhibited the BChE by 55.4% over 15.3% for AChE [63]. However, the amount of geraniol was not found to be a remarkable factor associated with the ChE inhibitory activity, in our investigations.

It seems that the presence of δ-cadinene is also important for inducing BChE inhibitory activity as *S. mirzayanii* and *S. syriaca* EOs containing high levels of that compound, showed good activity against BChE. Also, the amount of mesitylene as one of the main components of *S. syriaca* EO (9.13%) was found to be important for higher BChE inhibitory activity. However, further study is needed to determine whether these compounds are responsible for BChE inhibitory property.

On the other hand, no correlation was detected between the ChE inhibitory activity of the EOs and limonene, *trans*-β-caryophyllene, spathulenol, caryophyllene oxide, and linalool. Bonesi et al. studied the ChE inhibitory activity of *Cordia gilletii* De Wild. EO suggesting that *trans*-β-caryophyllene is playing an important role in BChE inhibitory activity [66]. Moreover, Sadaoui et al. suggested limonene as a strong BChE inhibitor (IC_50_ values of 51.6 and 66.7 µg/mL against AChE and BChE, respectively) [67]. However, the amount of these compounds was not found to be effective on the desired ChE inhibitory activity, in this study. As EOs of plants are complex mixtures, their inhibitory effects depend on the synergistic or antagonistic interactions.

According to the literature, AChE and BChE have approximately 65% amino acid sequence identity [68]. However, the substrate selectivity and sensitivity to the inhibitors of these enzymes, which are encoded by different genes, clearly vary. The primary distinction between the AChE and BChE relates to the acyl bonds and peripheral anionic sites where the substrate is linked to the enzyme. AChE has two aromatic amino acids, Phe295 and Phe297 in the acyl binding site whereas these amino acids have been replaced by linear ones, Leu286 and Val288 in the BChE [69]. The structural difference between these two enzyme active sites can explain why various compounds have different inhibitory activity. As stated earlier, AChE plays a greater role than BChE in the normal brain. However, in severe AD, the ratio between these two enzymes rises from 0.5 to 11 as the BChE effect grows [60]. Therefore, it is crucial to find a compound that could more efficiently inhibit the BChE. The acquired SI (selectivity index) from this study indicated that all the investigated EOs were able to inhibit the BChE more than the AChE, which makes them remarkable in treating AD in the advanced stages. Herein, the highest selectivity for BChE over AChE was obtained by *S. multicaulis* EO possessing high amounts of *trans*-β-caryophyllene (19.02%), borneol (12.52%), α-pinene (9.43%), and carvacrol (5.66%).

Comparing wild plants with cultivated ones, demonstrated that cultivated counterparts offer numerous advantages to the pharmaceutical industry, including fewer chemical changes, a more manageable supply chain, minimized batch variations, and stable raw material prices, albeit still higher than those for wild plants [70]. Because of factors such as changing climate conditions and depleting natural resources, traditional methods of growing aromatic plants do not always produce EOs with the appropriate quantity and quality of secondary metabolites. As a result, it is possible to optimize plant growth, EO efficiency, and EO constituents for cultivated herbs by using methods such as fertilizer or programmed temperature conditions and water supply.

In this work, the EO of *S. mirzayanii* cultivated in Shiraz, showed the strongest activity toward ChEs while the *S. spinosa* EO was the most potent inhibitor of α-glucosidase. It seems that they can be a potential and guaranteed source for the industrial production of new medicinal agents to control T2DM and AD.

## Conclusions

This study aimed to find natural and safe resources for the treatment of two common diseases; T2DM and AD, as many people with Type 2 diabetes have shown a higher risk of developing AD and anti-diabetic agents have been recently found to be active in the treatment of AD. Evaluation of 14 *Salvia* species EOs by GC–MS led to the identification of 139 compounds. The *S. spinosa* EO showed high inhibitory activity toward α-glucosidase, while three *S. mirzayanii* EOs exhibited the strongest inhibitory effects on ChEs. Plants in the genus *Salvia*, especially *S. mirzayanii* and *S. spinosa* which were cultivated in Shiraz, can be considered as natural resources for industrial production of important supplements to be effective as a treatment for AD and T2DM. However, it should be mentioned that further studies are required to determine the responsible bioactive components for desired biological activities. Also, the toxicology and bioavailability profile of EOs are in high demand.

## Data Availability

All relevant data are included within the manuscript. However, data are available from the corresponding author on reasonable requests.

## Abbreviations

ChE: Cholinesterase
EO: Essential oil
GC–MS: Gas chromatography-mass spectrometry
BChE: Butyrylcholinesterase
AChE: Acetylcholinesterase
DM: Diabetes Mellitus
AD: Alzheimer’s Disease
DMSO: Dimethyl sulfoxide
*p*NPG: *para*-Nitrophenol-α-D-glucopyranoside
DTNB: 5,5′-Dithio-bis-(2-nitrobenzoic acid)
ATCI: Acetylthiocholine iodide
BTCI: Butyrylthiocholine iodide
SI: Selectivity index
T2DM: Type 2 Diabetes Mellitus

## References

[1] Yadav A, Joshi A, Kothari S, Kachhwaha S, Purohit S (2017). Medicinal, nutritional and industrial applications of *Salvia* species: A revisit. Int J Pharm Sci Rev Res.

[2] Daniela T (1993). Salvia officinalis l. I Botanic characteristics, composition, use and cultivation. Cesk Farm.

[3] Bahadori MB, Salehi P, Sonboli A (2017). Comparative study of the essential oil composition of *Salvia urmiensis* and its enzyme inhibitory activities linked to diabetes mellitus and Alzheimer’s disease. Int J Food Prop.

[4] Karik U, Çinar O, Tuncturk M, Sekeroglu N, Gezici S (2018). Essential oil composition of some sage (Salvia spp.) species cultivated in İzmir (Turkey) ecological conditions. Indian J Pharm Educ.

[5] Sharifi-Rad M, Ozcelik B, Altın G, Daşkaya-Dikmen C, Martorell M, Ramírez-Alarcón K (2018). Salvia spp. plants-from farm to food applications and phytopharmacotherapy. Trends Food Sci Technol.

[6] Askari SF, Avan R, Tayarani-Najaran Z, Sahebkar A, Eghbali S (2021). Iranian *Salvia* species: a phytochemical and pharmacological update. Phytochemistry.

[7] Gad HA, Mamadalieva RZ, Khalil N, Zengin G, Najar B, Khojimatov OK (2022). GC-MS Chemical Profiling, Biological Investigation of Three *Salvia* Species Growing in Uzbekistan. Molecules.

[8] Lim Ah, Tock MJ, Kamatou GPP, Combrinck S, Sandasi M, Viljoen AM (2020). A chemometric assessment of essential oil variation of three Salvia species indigenous to South Africa. Phytochemistry.

[9] Temel HE, Demirci B, Demirci F, Celep F, Kahraman A, Doğan M (2016). Chemical characterization and anticholinesterase effects of essential oils derived from *Salvia* species. J Essent Oil Res.

[10] Villalta G, Salinas M, Calva J, Bec N, Larroque C, Vidari G (2021). Selective BuChE inhibitory activity, chemical composition, and enantiomeric content of the essential oil from Salvia leucantha Cav. collected in Ecuador. Plants.

[11] Menichini F, Tundis R, Loizzo MR, Bonesi M, Marrelli M, Statti GA (2009). Acetylcholinesterase and butyrylcholinesterase inhibition of ethanolic extract and monoterpenes from Pimpinella anisoides V Brig. (Apiaceae). Fitoterapia.

[12] Szwajgier D, Baranowska-Wójcik E (2019). Terpenes and phenylpropanoids as acetyl-and butyrylcholinesterase inhibitors: a comparative study. Curr Alzheimer Res.

[13] Kontogianni VG, Tomic G, Nikolic I, Nerantzaki AA, Sayyad N, Stosic-Grujicic S (2013). Phytochemical profile of *Rosmarinus officinalis* and *Salvia officinalis* extracts and correlation to their antioxidant and anti-proliferative activity. Food Chem.

[14] Taarit MB, Msaada K, Hosni K, Hammami M, Kchouk ME, Marzouk B (2009). Plant growth, essential oil yield and composition of sage (Salvia officinalis L.) fruits cultivated under salt stress conditions. Ind Crops Prod.

[15] Majouli K, Besbes Hlila M, Hamdi A, Flamini G, Ben Jannet H, Kenani A (2016). Antioxidant activity and α-glucosidase inhibition by essential oils from Hertia cheirifolia (L.). Ind Crops Prod.

[16] Aschner P, Karuranga S, James S, Simmons D, Basit A, Shaw JE (2021). The International Diabetes Federation's guide for diabetes epidemiological studies. Diabetes Res Clin Pract.

[17] Mahnashi M, Alqahtani Y, Alyami B, Alqarni A, Ayaz M, Ghufran M (2022). Phytochemical Analysis, α-Glucosidase and Amylase Inhibitory, and Molecular Docking Studies on Persicaria hydropiper L. Leaves Essential Oils. Evid Based Complementary Altern Med.

[18] Chaudhury A, Duvoor C, Reddy Dendi VS, Kraleti S, Chada A, Ravilla R (2017). Clinical review of antidiabetic drugs: implications for type 2 diabetes mellitus management. Front Endocrinol.

[19] El-Nashar HA, Eldehna WM, Al-Rashood ST, Alharbi A, Eskandrani RO, Aly SH (2021). GC/MS Analysis of Essential Oil and Enzyme Inhibitory Activities of *Syzygium cumini* (Pamposia) Grown in Egypt: Chemical Characterization and Molecular Docking Studies. Molecules.

[20] Elyasi Ghahfarrokhi A, Saeedi M, Khanavi M, Mojtabavi S, Kobarfard F, Faramarzi M (2022). Chemical composition and biological effects of Pistacia atlantica Desf. oleoresin essential oil. Res J Pharmacogn.

[21] Mechchate H, Es-Safi I, Haddad H, Bekkari H, Grafov A, Bousta D (2021). Combination of Catechin, Epicatechin, and Rutin: optimization of a novel complete antidiabetic formulation using a mixture design approach. J Nutr Biochem.

[22] Belhadj S, Hentati O, Hammami M, Ben Hadj A, Boudawara T, Dammak M (2018). Metabolic impairments and tissue disorders in alloxan-induced diabetic rats are alleviated by Salvia officinalis L. essential oil. Biomed Pharmacother.

[23] Assaggaf HM, Naceiri Mrabti H, Rajab BS, Attar AA, Alyamani RA, Hamed M (2022). chemical analysis and investigation of biological effects of Salvia officinalis essential oils at three phenological stages. Molecules.

[24] Savelev SU, Okello EJ, Perry EK (2004). Butyryl-and acetyl-cholinesterase inhibitory activities in essential oils of *Salvia* species and their constituents. Phytother Res.

[25] Zhang XX, Tian Y, Wang ZT, Ma YH, Tan L, Yu JT (2021). The epidemiology of Alzheimer’s disease modifiable risk factors and prevention. J Prev Alzheimer's Dis.

[26] Dabaghian F, Azadi A, Zarshenas MM (2022). Design, reformulation, and standardization of a traditional-based memory enhancer herbal preparation originated from Persian medicine. J Medicinal Plants.

[27] Moss M, Rouse M, Moss L (2014). Aromas of *Salvia* Species Enhance Everyday Prospective Memory Performance in Healthy Young Adults. Adv Chem Engineer Sci.

[28] Kennedy DO, Dodd FL, Robertson BC, Okello EJ, Reay JL, Scholey AB (2011). Monoterpenoid extract of sage (*Salvia lavandulaefolia*) with cholinesterase inhibiting properties improves cognitive performance and mood in healthy adults. J Psychopharmacol.

[29] Perry NSL, Houghton PJ, Jenner P, Keith A, Perry EK (2002). *Salvia lavandulaefolia* essential oil inhibits cholinesterase in vivo. Phytomedicine.

[30] Kivrak İ, Duru ME, Öztürk M, Mercan N, Harmandar M, Topçu G (2009). Antioxidant, anticholinesterase and antimicrobial constituents from the essential oil and ethanol extract of *Salvia potentillifolia*. Food Chem.

[31] Laborda R, Manzano I, Gamón M, Gavidia I, Perez-Bermudez P, Boluda R (2013). Effects of *Rosmarinus officinalis* and *Salvia officinalis* essential oils on Tetranychus urticae Koch (Acari: Tetranychidae). Ind Crops Prod.

[32] Bisio A, Ciarallo G, Romussi G, Fontana N, Mascolo N, Capasso R (1998). Chemical composition of essential oils from some *Salvia* species. Phytother Res.

[33] Lynn J, Park M, Ogunwale C, Acquaah-Mensah GK (2022). A tale of two diseases: Exploring mechanisms linking diabetes mellitus with Alzheimer’s disease. J Alzheimer's Dis.

[34] The NIST Database: https://webbook.nist.gov/chemistry/. Accessed 15 Mar 2022.

[35] Adams R (2005). Identification of Essential Oil Components by Gas Chromatography/Quadrupole Mass Spectroscopy. Carol Stream.

[36] Ellman GL, Courtney KD, Andres V, Featherstone RM (1961). A new and rapid colorimetric determination of acetylcholinesterase activity. Biochem Pharmacol.

[37] Rajabi Z, Ebrahimi M, Farajpour M, Mirza M, Ramshini H (2014). Compositions and yield variation of essential oils among and within nine *Salvia* species from various areas of Iran. Ind Crops Prod.

[38] Bilel H, Boubakri L, Zagrouba F, Hamdi N (2015). Chemical composition, antimicrobial and antioxidant activities of the essential oils from flowers of Salvia Sharifii. Eur J Org Chem.

[39] Aghaee Z, Alizadeh A, Honarvar M, Babadaei Samani R (2022). Phytochemical screening and antimicrobial activity of Salvia sharifii Rech. & Esfand from Iran. Nat Prod Res.

[40] Raafat K, Habib J (2018). Phytochemical Compositions and Antidiabetic Potentials of Salvia sclarea L. Essential Oils. J Oleo Sci.

[41] Shanaida M, Hudz N, Białoń M, Kryvtsowa M, Svydenko L, Filipska A (2021). Chromatographic profiles and antimicrobial activity of the essential oils obtained from some species and cultivars of the Mentheae tribe (Lamiaceae). Saudi J Biol Sci.

[42] Pitarokili D, Tzakou O, Loukis A (2006). Essential oil composition of Salvia verticillata, S. verbenaca, S. glutinosa and S. candidissima growing wild in Greece. Flavour Fragr J.

[43] Karamian R, Asadbegy M, Pakazad R (2014). Essential Oil Compositions, Antioxidant and Antibacterial Activities of Two Salvia Species (S. grossheimii Bioss. and S. syriaca L.) Growing in Iran. J Essent Oil Bear Plants.

[44] Arslan Ş, Kocabıyık K, Mutlu D, Semiz G (2021). Assessment of Cytotoxic and Apoptotic Effects of Salvia syriaca L. in Colorectal Adenocarcinoma Cell Line (Caco-2). Iran J Pharm Res.

[45] Bahadori MB, Valizadeh H, Farimani M (2016). Chemical Composition and Antimicrobial Activity of the Volatile Oil of *Salvia santolinifolia* Boiss. From Southeast of Iran. Pharm Sci.

[46] Fattahi B, Nazeri V, Kalantari S, Bonfill M, Fattahi M (2016). Essential oil variation in wild-growing populations of Salvia reuterana Boiss. collected from Iran: Using GC-MS and multivariate analysis. Ind Crops Prod.

[47] Talebi SM, Askary M, Khalili N, Matsyura A, Ghorbanpour M, Kariman K (2021). Genetic structure and essential oil composition in wild populations of *Salvia multicaulis* Vahl. Syst Ecol.

[48] Gharenaghadeh S, Karimi N, Forghani S, Nourazarian M, Gharehnaghadeh S, Jabbari V (2017). Application of Salvia multicaulis essential oil-containing nanoemulsion against food-borne pathogens. Food Biosci.

[49] Bahadori MB, Valizadeh H, Asghari B, Dinparast L, Moridi Farimani M, Bahadori S (2015). Chemical composition and antimicrobial, cytotoxicity, antioxidant and enzyme inhibitory activities of *Salvia spinosa* L. J Funct Foods.

[50] Flamini G, Cioni PL, Morelli I, Bader A (2007). Essential oils of the aerial parts of three Salvia species from Jordan: Salvia lanigera, S. spinosa and S. syriaca. Food Chem.

[51] Al-Jaber HI, Al-Qudah MA, Barhoumi LM, Abaza IF, Afifi FU (2012). Essential oil composition of the aerial parts of fresh and air-dried Salvia palaestina Benth. (Lamiaceae) growing wild in Jordan. Nat Prod Res.

[52] Baharfar R, Tajbakhsh M, Azimi R, Khalilzadeh MA, Eslami B (2009). Chemical Constituents of Essential Oils From the Leaves, Stems and Aerial Parts of Salvia virgata Jacq. From Iran. J Essent Oil Res.

[53] Sonboli A, Salehi P, Gharehnaghadeh S (2016). Chemical variability in the essential oil composition of *Salvia hypoleuca*, an endemic species from Iran. J Essent Oil Res.

[54] Nickavar B, Mojab F, Asgarpanah J (2005). Volatile composition of the essential oil of *Salvia hypoleuca* Benth. Int J Aromather.

[55] Asadollahi M, Firuzi O, Heidary Jamebozorgi F, Alizadeh M, Jassbi AR (2019). Ethnopharmacological studies, chemical composition, antibacterial and cytotoxic activities of essential oils of eleven *Salvia* in Iran. J Herb Med.

[56] Zomorodian K, Moein M, Pakshir K, Karami F, Sabahi Z (2017). Chemical composition and antimicrobial activities of the essential oil from *Salvia mirzayanii* leaves. J Evid Based Complementary Altern Med.

[57] Yamini Y, Khajeh M, Ghasemi E, Mirza M, Javidnia K (2008). Comparison of essential oil compositions of *Salvia mirzayanii* obtained by supercritical carbon dioxide extraction and hydrodistillation methods. Food Chem.

[58] Bahadori MB, Dinparast L, Zengin G, Sarikurkcu C, Bahadori S, Asghari B (2017). Functional components, antidiabetic, anti-Alzheimer’s disease, and antioxidant activities of *Salvia syriaca* L. Int J Food Prop.

[59] Hajlaoui H, Arraouadi S, Noumi E, Aouadi K, Adnan M, Khan MA (2021). Antimicrobial, Antioxidant, Anti-Acetylcholinesterase, Antidiabetic, and Pharmacokinetic Properties of Carum carvi L. and Coriandrum sativum L. Essential Oils Alone and in Combination. Molecules.

[60] Greig NH, Utsuki T, Yu QS, Zhu X, Holloway HW, Perry T (2001). A New Therapeutic Target in Alzheimer's Disease Treatment: Attention to Butyrylcholinesterase. Curr Med Res Opin.

[61] Akdeniz M, Yener I, Yilmaz MA, Irtegun Kandemir S, Tekin F, Ertas A (2021). A potential species for cosmetic and pharmaceutical industries: Insight to chemical and biological investigation of naturally grown and cultivated *Salvia multicaulis* Vahl. Ind Crops Prod.

[62] Kunduhoğlu B, Kurkcuoglu M, Duru M, Baser KHC (2011). Antimicrobial and anticholinesterase activities of the essential oils isolated from Salvia dicroantha Stapf., Salvia verticillata L. subsp. amasiaca (Freyn and Bornm.) Bornm. and Salvia wiedemannii Boiss. J Med Plant Res.

[63] Orhan I, Kartal M, Kan Y, Sener B (2008). Activity of essential oils and individual components against acetyl- and butyrylcholinesterase. Z Naturforsch C J Biosci.

[64] Loizzo MR, Menichini F, Tundis R, Bonesi M, Conforti F, Nadjafi F (2009). In vitro biological activity of Salvia leriifolia Benth. essential oil relevant to the treatment of Alzheimer’s disease. J Oleo Sci.

[65] Chowdhury S, Kumar S (2020). Alpha-terpinyl acetate: A natural monoterpenoid from *Elettaria cardamomum* as multi-target directed ligand in Alzheimer’s disease. J Funct Foods.

[66] Bonesi M, Okusa PN, Tundis R, Loizzo MR, Menichini F, Stévigny C (2011). Chemical composition, antioxidant properties and anti-cholinesterase activity of Cordia gilletii (Boraginaceae) leaves essential oil. Nat Prod Commun.

[67] Sadaoui N, Bec N, Barragan-Montero V, Kadri N, Cuisinier F, Larroque C (2018). The essential oil of Algerian Ammodaucus leucotrichus Coss. & Dur. and its effect on the cholinesterase and monoamine oxidase activities. Fitoterapia.

[68] Lane RM, Potkin SG, Enz A. Targeting acetylcholinesterase and butyrylcholinesterase in dementia. Int J Neuropsychopharmacol. 2006;9(1):101–24. 10.1017/S1461145705005833 16083515

[69] Tundis R, Leporini M, Bonesi M, Rovito S, Passalacqua NG. *Salvia officinalis* L. from Italy: A comparative chemical and biological study of its essential oil in the mediterranean context. Molecules. 2020;25(24):5826. 10.3390/molecules25245826 PMC7763040 33321838

[70] Lubbe A, Verpoorte R. Cultivation of medicinal and aromatic plants for specialty industrial materials. Ind crops prod. 2011;34(1):785–801.

